# Supplementary material for: Observed positive feedback between surface ablation and crevasse formation drives glacier acceleration and potential surge
Source: Nat Commun. 2025 Dec 18;16:11227. doi: 10.1038/s41467-025-66349-9 (PMC12714843; doi:10.1038/s41467-025-66349-9)
Supplement: Supplementary file 1 — Supplementary Information [file 41467_2025_66349_MOESM1_ESM.pdf]

Supplementary materials of Observed positive feedback between surface ablation and crevasse formation drives glacier acceleration and potential surge

Ugo Nanni[1,*], Coline Bouchayer[1,2], Henning Åkesson[1], Pierre-Marie Lefeuvre[3,4], Erik S. Mannerfelt[1], Andreas Köhler[5], Oliver Gagliardini[6], Louise S. Schmidt[1], John Hult[1], François Renard[2,7], Thomas V. Schuler[1]

[1]Department of Geosciences, University of Oslo, Oslo, Norway
[2]Njord Centre, Departments of Geosciences and Physics, University of Oslo, Oslo, Norway
[3]Norwegian Polar Institute, Tromsø, Norway
[4]Norwegian Meteorological Institute, Oslo, Norway
[5]NORSAR, Kjeller, Norway
[6]IGE, Université Grenoble Alpes, Université Savoie Mont Blanc, CNRS, IRD, Université Gustave Eiffel, Grenoble, France
[7]ISTerre, Université Grenoble Alpes, Université Savoie Mont Blanc, CNRS, IRD, Université Gustave Eiffel, Grenoble, France

*Corresponding author: ugo.nanni0158@gmail.com

5.1 Glacier surface velocity

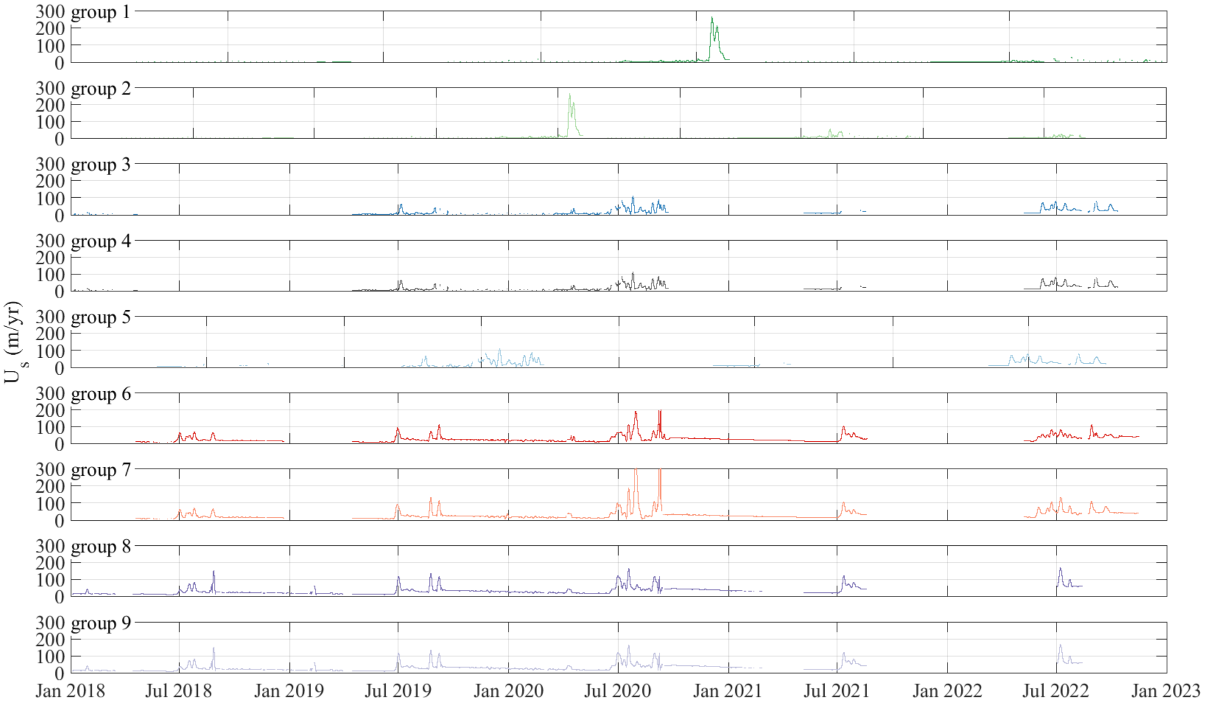

Supplementary Figure 1: **Glacier surface velocity measured with the Global Navigation Satellite System stations.** Each row corresponds to the glacier surface velocity measured at the location at each of the group, from 1 (top row, glacier front) to 9 (bottom row, glacier upper part). Starting from January 2018, ending in December 2023.

5.2 Runoff, Subglacial hydraulic properties, Seismic power and Icequake activity

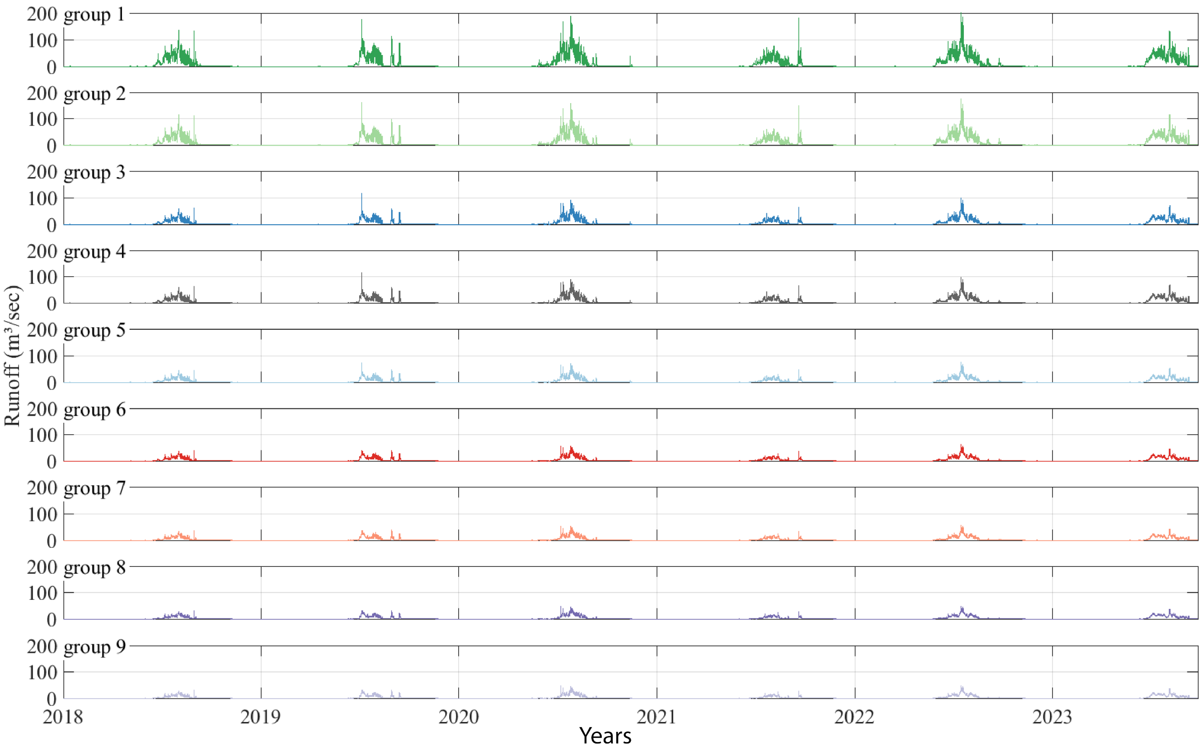

Supplementary Figure 2: **Timeseries of Runoff modeled for each of the nine locations.** Each row corresponds to the runoff calculated at the location at each of the group, from 1 (top row, glacier front) to 9 (bottom row, glacier upper part). Starting from January 2018, ending in December 2023.

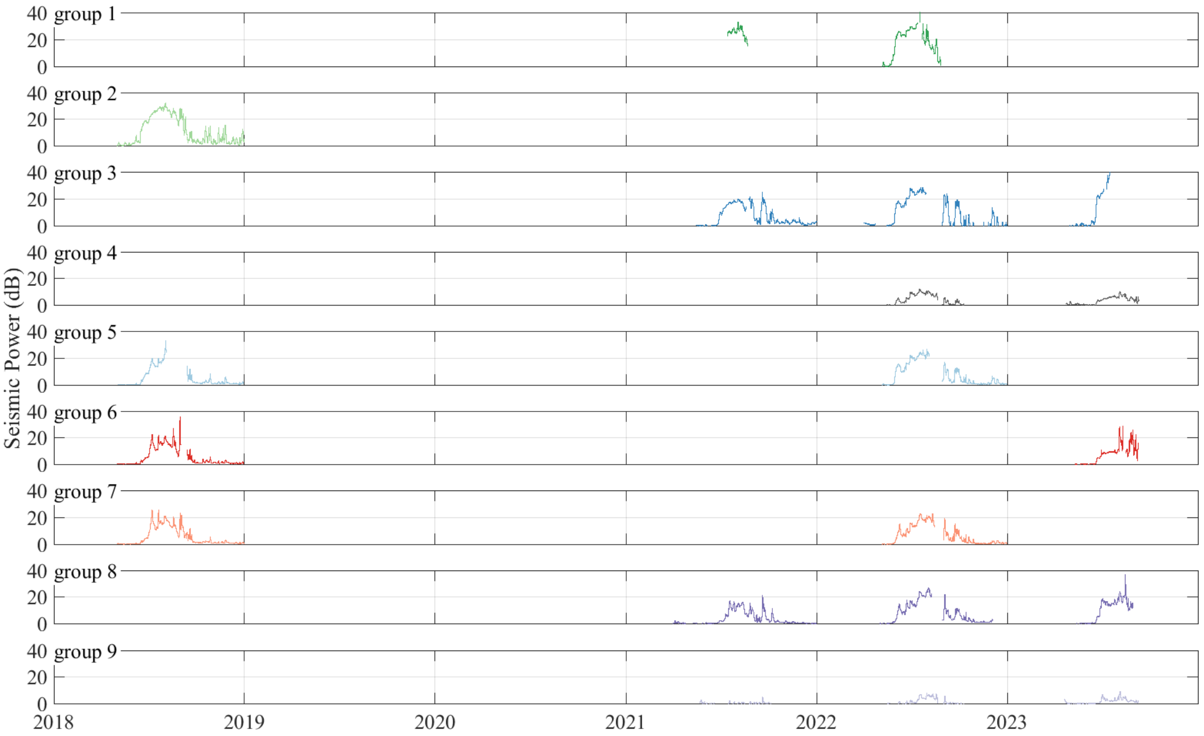

Supplementary Figure 3: **Seismic power averaged in the [5-10] Hz band**. Each row corresponds to the Seismic power averaged in the [5-10] Hz band measured at the location at each of the group, from 1 (top row, glacier front) to 9 (bottom row, glacier upper part). Starting from January 2018, ending in December 2023. The black line show the moving average over 1 day. The power is shown relative to 0, the minimum power at each station.

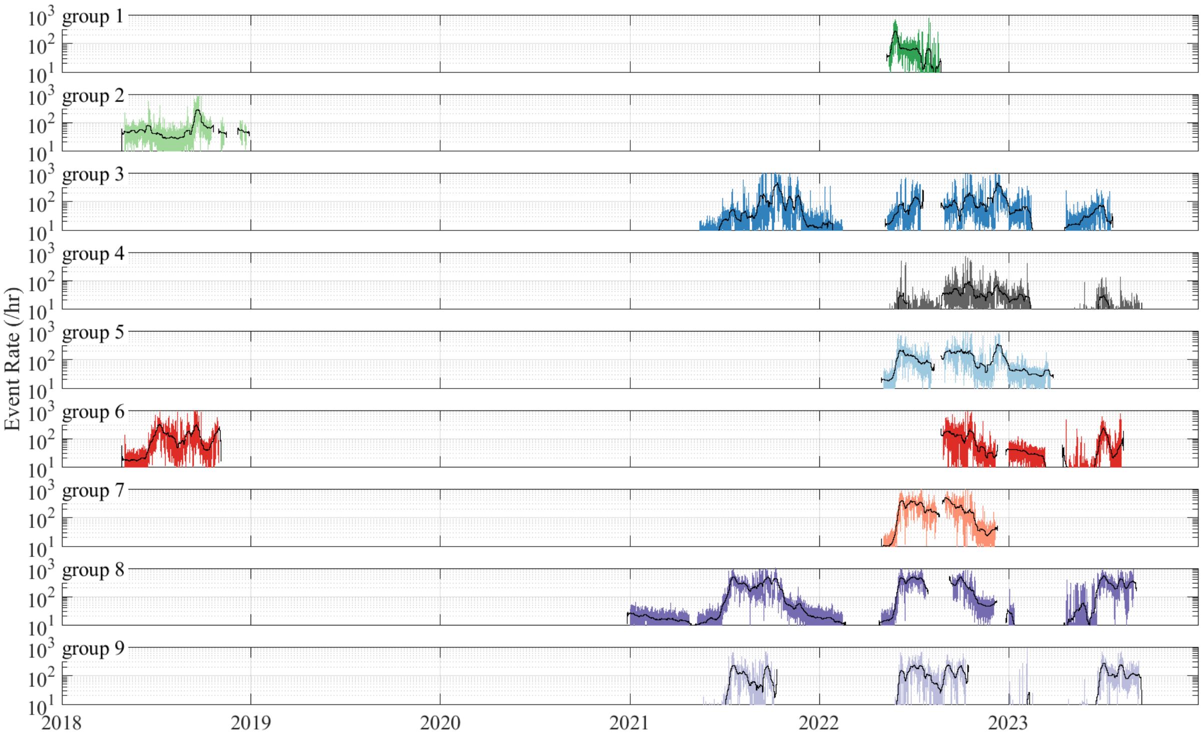

Supplementary Figure 4: **Event rate**. Each row corresponds to the event rate measured at the location at each of the group, from 1 (top row, glacier front) to 9 (bottom row, glacier upper part). Starting from January 2018, ending in December 2023. The black line show the moving average over 1 day.

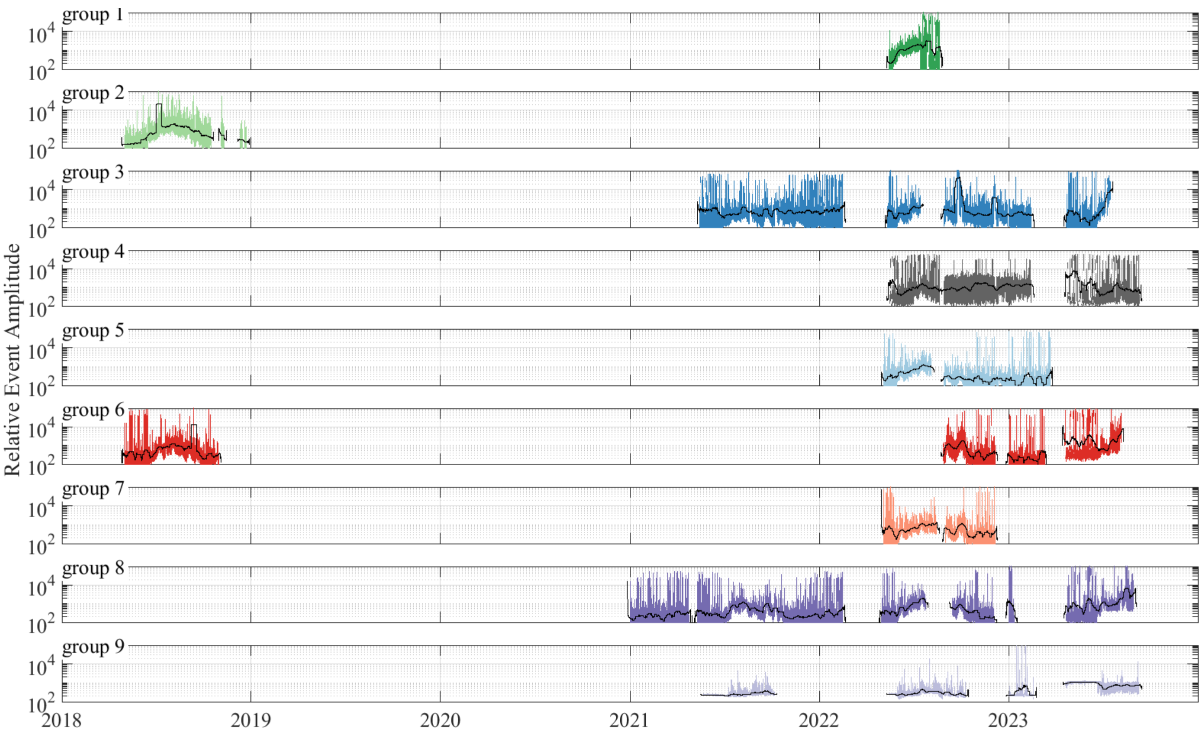

Supplementary Figure 5: **Event amplitude**. Each row corresponds to the event amplitude measured at the location at each of the group, from 1 (top row, glacier front) to 9 (bottom row, glacier upper part). Starting from January 2018, ending in December 2023. The black line show the moving average over 1 day. Event amplitude is relative to winter mean amplitude and is in count.
